# Evolving epidemiology of pneumocystis pneumonia: Findings from a longitudinal population-based study and a retrospective multi-center study in Germany

**DOI:** 10.1016/j.lanepe.2022.100400

**Published:** 2022-05-15

**Authors:** Benedikt Kolbrink, Jubin Scheikholeslami-Sabzewari, Christoph Borzikowsky, Friedrich A. von Samson-Himmelstjerna, Andrew J. Ullmann, Ulrich Kunzendorf, Kevin Schulte

**Affiliations:** a Department of Nephrology and Hypertension, University Hospital Schleswig-Holstein, Christian-Albrechts-University, Arnold-Heller-Str. 3 Haus C, Kiel 24105, Germany; b Institute of Medical Informatics and Statistics, Christian-Albrechts-University, Kiel, Germany; c Department for Internal Medicine II, University Hospital Würzburg, Würzburg, Germany

**Keywords:** Pneumocystis pneumonia, Epidemiology, Opportunistic infection, Immunosuppression, HIV, Non-HIV, AIDS, acquired immunodeficiency syndrome, DRG, diagnosis related groups, GFSO, german federal statistical office, HIV, human immunodeficiency virus, ICD, international classification of diseases, ICU, intensive care unit, IQR, interquartile range, PCP, pneumocystis pneumonia

## Abstract

**Background:**

Pneumocystis pneumonia (PCP) is a life-threatening opportunistic infectious disease of immunocompromised patients. Its incidence has decreased worldwide in the past, but data concerning its recent epidemiology are lacking.

**Methods:**

We retrospectively analyzed all German inpatient cases from January 1, 2014 to December 31, 2019, to describe the recent epidemiology, incidence, clinical course, mortality and underlying diseases of PCP. Simultaneously, we conducted a retrospective multi-center study at two German university hospitals, and analyzed PCP cases treated there to gain deeper insights on the basis of primary patient data.

**Findings:**

The incidence of PCP significantly increased from 2·3 to 2·6 per 100,000 population from 2014 to 2019 (1,857 to 2,172 cases, +17·0%, *p* < 0·0001), as well as PCP-related deaths (516 to 615 cases, +19·2%, *p* = 0·011). The spectrum of underlying diseases changed: Risk groups with established chemoprophylaxis for PCP based on international guidelines (HIV, hematologic malignancies, and transplantation) showed a significant decrease in PCP cases and deaths. Others, especially those with solid malignancies, and autoimmune and pulmonary diseases showed a significant increase in case numbers and deaths. Data from the retrospective multi-center study added information regarding prophylaxis and diagnostics of PCP.

**Interpretation:**

The incidence of PCP has reversed its trend, showing a significant increase in mortality on population level. Patients who were not previously considered in prophylactic measures are increasingly affected by PCP. This development deserves further investigation, and additional comprehensive guidelines for the use of chemoprophylaxis in new risk groups are needed.

**Funding:**

Department of Nephrology and Hypertension, University Hospital Schleswig-Holstein, Kiel.

**Research in context**

**Evidence before this study**

We searched PubMed for articles published between January 1, 2000, and June 30, 2021, with no language restrictions on the epidemiology of pneumocystis pneumonia (PCP) in patients with the human immunodeficiency virus (HIV) and non-HIV patients, and recommendations for prophylaxis. We used the following search terms: “pneumocystis pneumonia” OR “pneumocystosis” AND “epidemiology” OR “incidence” OR “prophylaxis” AND “HIV” OR “non-HIV”. Since PCP is a major acquired immunodeficiency syndrome (AIDS)-defining disease, numerous reports have focused on HIV patients, which have shown a decline in the incidence of PCP worldwide, from the mid-1990s through 2010. In contrast, only three longitudinal studies on the epidemiology of PCP in the general populations of industrialized countries (USA, Spain and France) have published data from 2000 through 2014. They reported a further decrease in the incidence and deaths of overall- and HIV-specific PCP. Since no epidemiologic data later than 2014 have been published, it is unclear how the incidence, particularly that of non-HIV patients, has evolved in recent years.

**Added value of this study**

Our study is the first to describe an increase in the incidence of PCP in the general population since 2000. This comeback of PCP is solely due to an increase in PCP cases among non-HIV risk groups, while the number of HIV-related PCP cases continues to decline slightly. We report a substantial increase in PCP-related deaths at the population level, which is a new and concerning finding. Furthermore, we describe a remarkable trend: Where chemoprophylaxis for PCP is established based on international guidelines (namely, for HIV- and hematologic patients and organ transplant recipients), case numbers and mortality rates are decreasing. In contrast, they are increasing significantly in other risk groups (namely, patients with oncological, rheumatological and pulmonological diseases). Finally, we demonstrate that mortality of these patients is very high, but use of PCP prophylaxis is inadequate, implying that better use of PCP prophylaxis is of extraordinary importance in these groups.

**Implications of all the available evidence**

The overall incidence and number of PCP-related deaths have increased significantly in recent years, mainly among oncological, rheumatological and pulmonological patients. However, the opposite trend has been observed in patient groups for which there already are guideline recommendations for PCP prophylaxis. Further research and new guidelines for PCP prophylaxis in previously neglected patient groups are necessary.

Alt-text: Unlabelled box

## Introduction

Pneumocystis pneumonia (PCP) is an opportunistic pulmonary infection caused by the fungus *Pneumocystis jirovecii.* It has traditionally been considered an acquired immunodeficiency syndrome (AIDS)-defining illness, although it also occurs in immunosuppressed patients without the human immunodeficiency virus (HIV), where it more often takes a fatal course.^1^, ^2^, ^3^, ^4^ Despite the existence of effective chemoprophylaxis, current global estimates are as high as 500,000 annual cases, with a mortality of 10 to 30%.^5^ To optimize current prevention strategies, a precise knowledge of PCP's epidemiology is of central importance; however, studies providing data on the epidemiology of PCP have been scarce in recent years.

Based on the international literature, the incidence of PCP, especially in HIV patients, has declined since the 1990s. In addition to significant improvements in HIV detection and the wide availability of antiretroviral therapy, the establishment of prophylaxis for PCP as a standard HIV therapy has played a decisive role.^1^^,^^2^^,^^6^, ^7^, ^8^ However, the more recent epidemiology of PCP is difficult to assess. The current incidence and/or number of PCP-related deaths in the general population have been reported in only three longitudinal studies from France, Spain and the US, and their data extended at most to 2014. These studies showed a further pronounced decline in PCP incidence and deaths, which had stalled in recent years.^9^, ^10^, ^11^ This picture is challenged by some smaller studies that have shown an increase in the incidence and mortality rates.^12^^,^^13^ In addition, there are a multitude of center-based studies focusing on PCP in patients with very specific underlying diseases, but these investigations cannot provide meaningful insights into the epidemiology of PCP due to their small cohorts and selection bias.

Taking into consideration the declining relevance of HIV-related PCP and the worldwide rise in the number of people with risk factors for non-HIV-related PCP – such as old age, cancer, and immunosuppression – it seems possible that the dynamics of PCP are changing unobserved.^14^^,^^15^ Therefore, current epidemiological data are essential to identify patient populations in which treatment could be improved through consistent primary prophylaxes or lower thresholds for diagnostics and therapies. To address this problem, we combined secondary data of all German inpatient cases with data from a retrospective multi-center study conducted in two German university hospitals to address the following questions:

1. How frequent is PCP currently and is its incidence increasing?
2. Which underlying diseases are responsible for the development of PCP?
3. What are the course and outcomes of PCP?

## Materials and methods

### Data source and study design of the nationwide analysis of PCP inpatient cases

The data source for this nationwide analysis was the Statistisches Bundesamt (which is the German Federal Statistical Office, GFSO). All hospitals in Germany are required by law to report annual data on all inpatient cases to the diagnosis related group (DRG) database of the GFSO, the dataset therefore has full coverage of German inpatient cases. The original purpose of collecting these data is to determine hospital charges for subsequent years under the German DRG system. Our study is therefore a secondary data analysis using data that were not primarily collected for scientific purposes. The reported data mandatorily include basic patient and hospital characteristics and one main diagnosis. Further included can be up to 89 subsidiary diagnoses according to the International Classification of Diseases (ICD) 10^th^ Revision, German Modification, as well as up to 100 German Operation and Procedure Classification System key codes per individual case. Specific anonymized data can be requested for research purposes via remote data processing; therefore, no ethics approval is required.

The GFSO enabled us to analyze data for the years 2014 to 2019, which was the most current information available at the time the research was conducted (October 2021). Among all the German inpatient cases during this period, we identified those with the main or one of the subsidiary diagnoses of an ICD-code for PCP (B59 until 2018, changed to B48.5 in Germany in 2019). Additional detailed information was retrieved from these cases: age, sex, reason for discharge, information regarding clinical course, procedures performed (Table S1), and the underlying disease leading to immunosuppression. Patients treated at university hospitals were identified by hospital institution codes.

To identify the causes of PCP, we formed the following groups based on various ICD-codes (Table S2): (1) HIV and AIDS, (2) solid malignancies, (3) hematologic malignancies, (4) other hematologic diseases, (5) rheumatic/kidney diseases, (6) pulmonary diseases, (7) gastrointestinal diseases, and (8) solid organ transplant. Patients with more than one possible risk factor for developing PCP were classified in the group “more than one underlying disease”, whereas patients without any identifiable risk factors were assigned to the group “not otherwise classified”. Based on this classification, every patient was assigned to exactly one group and there were no duplications of patients among different groups (Figure S1). We used annual census data from the GFSO to calculate the overall and sex-specific incidence of PCP.^16^

### Study design of the retrospective multi-center study

Simultaneously, we conducted a retrospective cohort study of all PCP inpatient cases that had been treated between 2014 and 2019 at the University Hospitals of Kiel and Lübeck, two northern German tertiary care centers. All inpatient cases in the internal databases from 2014 to 2019 were screened for the presence of PCP in their diagnoses at discharge. Among the cases identified in this way, all other necessary information was collected by chart review and anonymized for further analyses. None of the patients had been hospitalized twice for PCP during the study period. This part of the study was approved by the responsible ethics committee of the Christian-Albrechts-University of Kiel (file number D600/20).

### Statistical methods

Quantitative results are reported as absolute and relative frequencies or medians with interquartile ranges (IQR), as appropriate. The chi-square test or Fisher's exact test were used to compare dichotomous variables as well as changes in the number of cases or deaths between the first and the last year of the study, depending on the sample size. Time to pathogen detection and time to therapy between the different groups were not normally distributed, therefore the non-parametric Mann-Whitney U-test was applied for statistical testing. Results were considered statistically significant with a *p*-value <0·05. Syntax files were created in IBM SPSS Statistics for Windows (version 22.0.0.2, IBM, 2013) to analyze the data provided by the GFSO. In each of these syntax files, the requested data set elements were checked for completeness. When querying the relative proportion of all PCP patients treated in university hospitals nationwide, 364 of the total 12,455 PCP cases could not be classified due to missing data; these cases had not been taken into account when the proportion of PCP cases in university hospitals nationwide was given. For all other variables examined, the data set of the GFSO was complete and all cases have been included in the analyses.

### Role of the funding source

The funding source of this study had no influence on the study's design, data collection, analysis, interpretation, writing of the paper, or the decision to submit the paper for publication.

## Results

### PCP incidence is increasing

**Figure 1:**
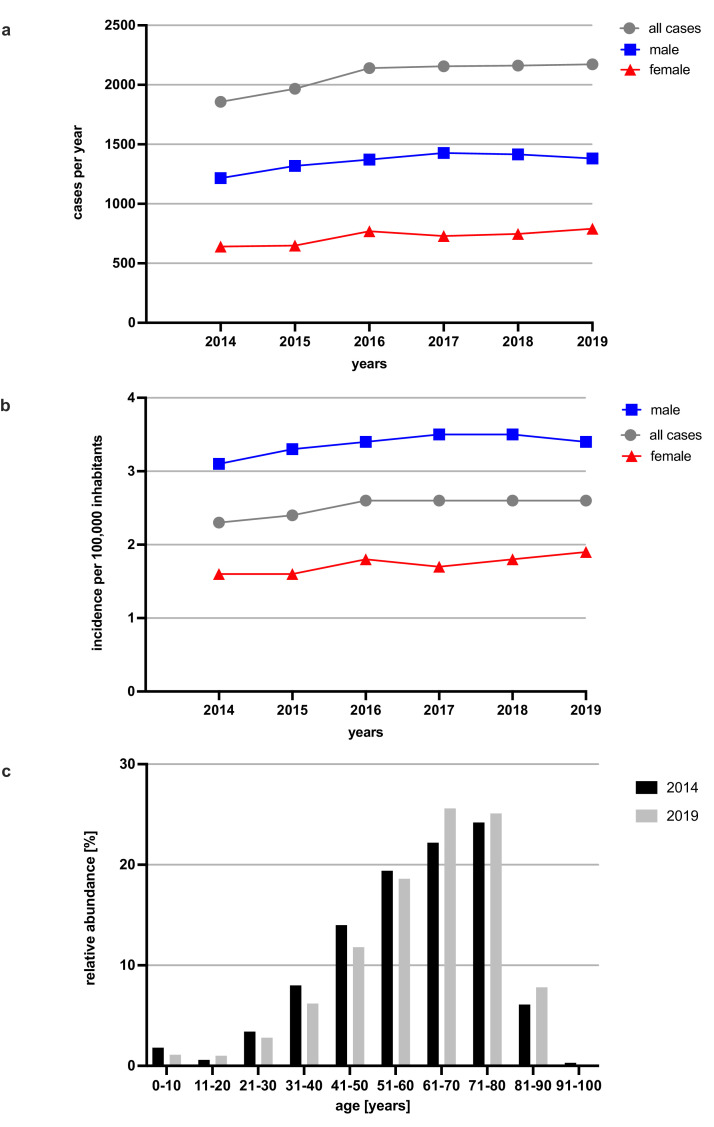
Epidemiological parameters of PCP in Germany 2014–2019. (a) Absolute numbers of PCP cases treated yearly in German hospitals (2014–2019), all (gray), male (blue) and female (red) patients; (b) Overall (gray) and sex-specific (red/blue) incidence of PCP in Germany (2014-2019); (c) Relative distribution of PCP patients by age in Germany in 2014 (black) and 2019 (gray).

A total of 112,640,154 inpatient cases were included in our nationwide secondary data analysis, of which 12,455 were identified as PCP cases (11·1 per 100,000 hospitalizations). We observed a continuous, significant increase in the annual number of PCP patients from 1857 in 2014 to 2172 in 2019 (+17·0%, *p* < 0·0001) (Table S3). The annual incidence increased from 2·3 to 2·6 per 100,000 inhabitants and approximately two-thirds (65·3%) of the patients were male (Figure 1A/B). Nearly half of PCP patients (48·8%) were between 50 and 75 years of age, with an increase in the median patient age from 62 (IQR 49–72) to 64 (IQR 52–74) years between 2014 and 2019 (Figure 1C). A total of 3655 (30·2%) PCP patients were treated in university hospitals nationwide.

### Underlying diseases are changing

**Figure 2:**
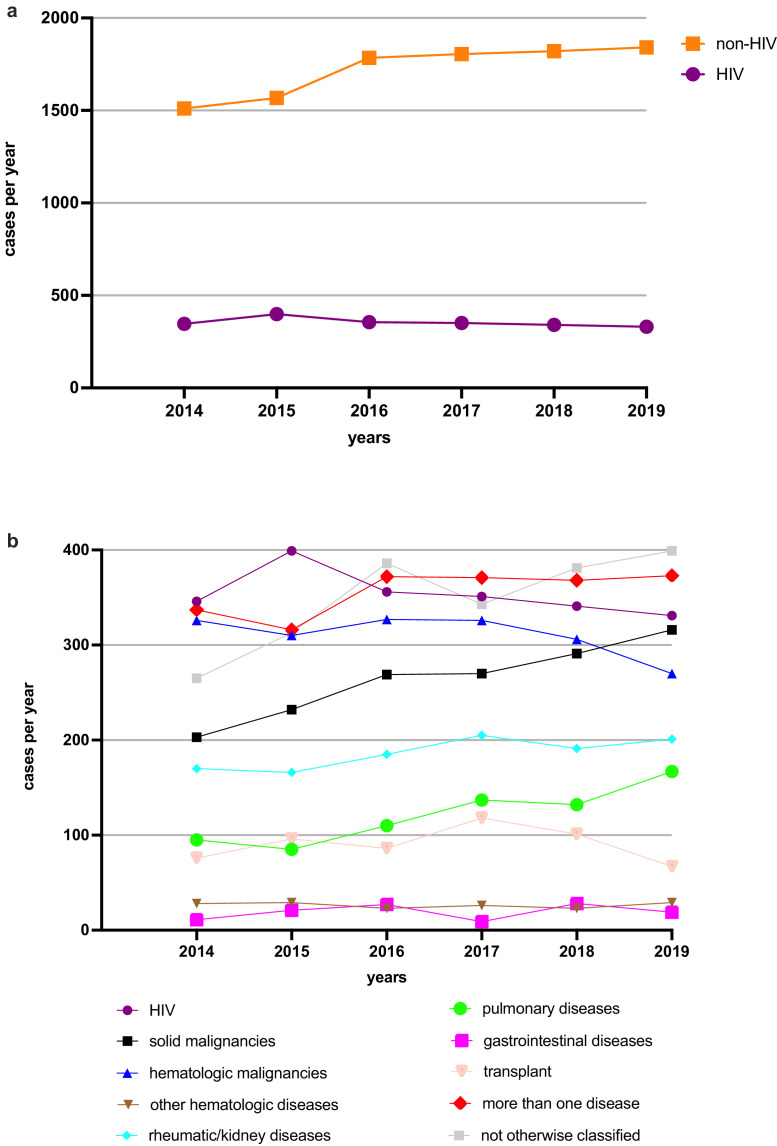
Underlying diseases of PCP patients are changing. (a) Absolute numbers of PCP cases yearly in HIV- (purple) and non-HIV patients (orange) from 2014 to 2019 in Germany; (b) Absolute numbers of PCP cases with pre-specified groups of underlying diseases.

Among all the PCP cases, 2124 (17·1%) occurred in HIV-seropositive patients and 10,331 (82·9%) in non-HIV patients. The number of annual HIV-related PCP cases decreased significantly from 346 in 2014 to 331 in 2019 (-4·3%, *p* = 0·0046), while during the same period, the number of non-HIV-related PCP cases increased from 1511 to 1,841 (+21·8%, *p* = 0·0046) (Figure 2A). Patients with HIV were younger (in 2019 the median age was 46 [IQR 37–53] years) than non-HIV patients (in 2019 the median age was 67 [IQR 57–75] years) (Figure S2). The distribution of underlying immunosuppressive diseases among the non-HIV patients changed significantly. There was a significant increase in the annual number of cases among the “pulmonary diseases” (95 to 167; +75·8%, *p* = 0·0010), "solid malignancies" (203 to 316; +55·7%, *p* = 0·0007), and “not otherwise classified” (265 to 399; +50·6%, *p* = 0·0005) groups. The groups with "more than one underlying disease" (337 to 373; +10·7%, *p* = 0·43) and "rheumatic/kidney diseases" (170 to 201; +18·2%, *p* = 0·96) showed positive, but not significant trends. However, the annual number of cases in the group with "hematologic malignancies" decreased significantly (326 to 270; -17·2%, *p* < 0·0001), whereas the “transplantation” group also showed a negative, but not significant trend (76 to 67; -11·8%, *p* = 0·088) (Figure 2B, Table S4).

### PCP-related deaths are increasing

**Figure 3:**
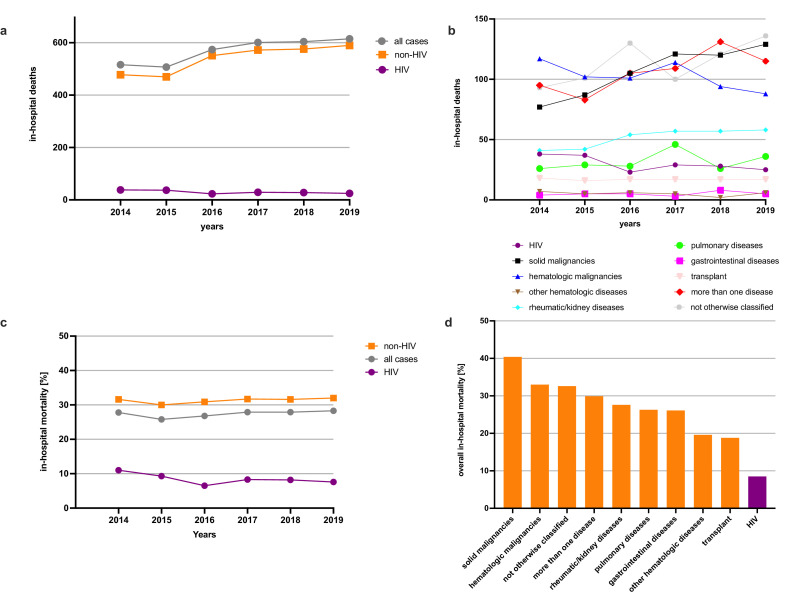
PCP-related deaths are increasing. (a) Absolute numbers of PCP-related in-hospital deaths yearly among all (gray), HIV- (purple) and non-HIV patients (orange) from 2014 to 2019 in Germany; (b) Absolute numbers of PCP-related in-hospital deaths among patients with pre-specified groups of underlying diseases; (c) Trends in the relative in-hospital mortality overall (gray), and HIV (purple) and non-HIV (orange) patients from 2014 to 2019; (d) Mean in-hospital mortality of all PCP cases with pre-specified groups of underlying diseases from 2014 to 2019.

A significant increase was found in PCP-related deaths from 516 cases in 2014 to 615 cases in 2019 (+19·2%, *p* = 0·011) (Figure 3A, Table S3). As PCP-related deaths in HIV patients significantly decreased from 38 to 25 per year (-34·2%, *p* = 0·019), this development was mainly due to an increase of deaths in non-HIV patients from 478 to 590 per year (+23·4%, *p* = 0·019). Pronounced changes were also observed in the number of annual deaths among the subgroups of non-HIV cases. We observed a significant increase in the annual number of deaths in the group "solid malignancies" (77 to 129; +67·5%, *p* = 0·0086). Positive trends were also obvious in the following groups: “not otherwise classified” (93 to 136; +46·2%, *p* = 0·11), "rheumatic/kidney diseases" (41 to 58; +41·5%, *p* = 0·40), “pulmonary diseases” (26 to 36; +38·5%, *p* = 0·6010) and "more than one underlying disease" (95 to 115; +21·1%, *p* = 0·94). On the contrary, the number of deaths decreased significantly in the "hematologic malignancies" (117 to 88; -24·8%, *p* = 0·0003) and slightly in the “transplant” (18 to 17; -5·6%, *p* = 0·50) groups (Figure 3B, Table S5).

Among all the underlying diseases, HIV had, by far, the lowest in-hospital mortality, which decreased from 11·0% in 2014 to 7·6% in 2019. However, the mean in-hospital mortality of non-HIV patients plateaued at high levels (31·6% in 2014 vs. 32·0% in 2019) (Figure 3C). Considering the in-hospital mortality of each non-HIV group over the entire study period, the group with “solid malignancies” had the highest (40·4%) mortality rate, and the “transplant” group had the lowest (18·8%) mortality rate (Figure 3D). It is noteworthy, with regard to the treatment and course of PCP, that cross-sectional imaging of the thorax and bronchoscopies were performed significantly less frequently for HIV patients than they were for non-HIV patients (Tables 1 and S6/7). Furthermore, all the parameters that were investigated indicated that a severe course of disease (the need for ICU care, mechanical ventilation, renal replacement therapy, organ failure) occurred substantially more often among non-HIV patients than it did among HIV patients (Tables 2 and S8).

**Table 1:** Diagnostic procedures performed on PCP patients (nationwide).

Procedure	All patients (*n* = 12,455)	HIV (*n* = 2,124)	Non-HIV (*n* = 10,331)	p-value HIV vs non-HIV
Thorax CT/MRI	8,932 (71·7%)	1,307 (61·5%)	7,625 (73·8%)	<0·0001†
Bronchoscopy	8,894 (71·4%)	1,440 (67·8%)	7,454 (72·2%)	0·0003†
Thorax CT/MRI and bronchoscopy	6,919 (55·6%)	1,013 (47·7%)	5,906 (57·2%)	<0·0001†

†Chi-square test.

PCP, pneumocystis pneumonia; CT, computed tomography; MRI, magnetic resonance imaging; HIV, human immunodeficiency virus.

**Table 2:** Course of PCP and its complications (nationwide).

Event	All cases (*n* = 12,455)	HIV (*n* = 2,124)	Non-HIV (*n* = 10,331)
Death	3,417 (27·4%)	180 (8·5%)	3,237 (31·3%)
ICU care	5,494 (44·1%)	705 (33·2%)	4,789 (46·4%)
Mechanical ventilation	5,142 (41·3%)	574 (27·0%)	4,568 (44·2%)
Renal replacement therapy	1,663 (13·4%)	106 (5·0%)	1,557 (15·1%)
Multiple organ dysfunction	5,023 (40·3%)	422 (19·9%)	4,601 (44·5%)
Palliative care	392 (3·1%)	0 (0·0%)	392 (3·8%)
Cardiac arrest/resuscitation	489 (3·9%)	37 (1·7%)	447 (4·3%)

PCP, pneumocystis pneumonia; HIV, human immunodeficiency virus; ICU, intensive care unit.

### In-depth analysis of primary data in the retrospective multi-center study

The retrospective multi-center study evaluated 627,970 inpatient cases from January 1, 2014 to December 31, 2019. A total of 68 PCP cases were identified, corresponding to a rate of 10·5 per 100,000 hospitalizations (Figure S3). Of these cases, patients had a median age of 56 years (IQR 44–69) and 50 (73·5%) were male. We identified 19 (27·9%) HIV patients and 49 (72·1%) non-HIV patients. Eleven (16·2%) of the non-HIV patients were in the “transplant” group, 9 (13·2%) in the group with “hematologic malignancies”, 8 (11·8%) in the group with “more than one underlying disease”, 7 (10·3%) in each of the “solid malignancies” and “rheumatic/kidney diseases” groups, 3 (4·4%) in the group “not otherwise classified”, 2 (2·9%) in the group with “other hematologic diseases”, and 1 (1·5%) each of the “pulmonary diseases” and “gastrointestinal diseases” groups (Figure S4). Of the 68 cases, 15 (22·1%) had a fatal outcome, with mortality being substantially higher among non-HIV patients than it was among HIV patients (28·6% vs. 5·3%) (Tables 3 and S9). Remarkably, pathogen detection was successful in a significantly lower proportion of non-HIV patients than it was in HIV patients (73·5% vs. 100%, *p* = 0·014). In addition, therapy was started significantly later in the non-HIV patients, although the median time to pathogen detection did not differ (Table 4). Furthermore, although only 4 (8·2%) of the non-HIV patients did not have an immunosuppressive condition in their medical history, 45 (91·8%) were not on prescribed chemoprophylaxis at the time they developed PCP (Table 5).

**Table 3:** Course of PCP and its complications (multi-center study).

Event	All cases (*n* = 68)	HIV (*n* = 19)	Non-HIV (*n* = 49)
Death	15 (22·1%)	1 (5·3%)	14 (28·6%)
ICU	25 (36·8%)	5 (26·3%)	20 (40·8%)
Mechanical ventilation	41 (60·3%)	8 (42·1%)	33 (67·3%)
Renal replacement therapy	17 (25·0%)	1 (5·3%)	16 (32·7%)
Multiple organ dysfunction	41 (60·3%)	10 (52·6%)	31 (63·3%)
Palliative care	7 (10·3%)	1 (5·3%)	6 (12·2%)
Cardiac arrest/resuscitation	0 (0·0%)	0 (0·0%)	1 (2·0%)

PCP, pneumocystis pneumonia; HIV, human immunodeficiency virus; ICU, intensive care unit.

**Table 4:** Diagnostic procedures and time to therapy (multi-center study).

Procedures	All cases (*n* = 68)	HIV (*n* = 19)	Non-HIV (*n* = 49)	*p*-value HIV vs. non-HIV
Thorax CT/MRI	55 (80·9%)	12 (63·2%)	43 (87·8%)	0·036†
Bronchoscopy	61 (89·7%)	17 (89·5%)	44 (89·8%)	1†
Thorax CT/MRI and bronchoscopy	51 (75·0%)	12 (63·2%)	39 (79·6%)	0·21†
Pathogen detection successful*	55 (80·9%)	19 (100·0%)	36 (73·5%)	0·014†
Time to pathogen detection [median number of days (IQR)]**	3 (1;7)	3 (0;7)	3 (2;8)	0·38‡
Time to therapy [median number of days (IQR)]	3 (0;7)	1 (0;4)	3 (1;9)	0·019‡

^†^ Fisher's exact test.

^‡^ Mann-Whitney U-test.

*Direct detection of *Pneumocystis jirovecii* via PCR, microscopy or immunofluorescence.

**Only patients with successful pathogen detection were included in this analysis.

CT, computed tomography; MRI, magnetic resonance imaging; HIV, human immunodeficiency virus; IQR, interquartile range.

**Table 5:** Use of PCP prophylaxis (multi-center study).

Circumstances regarding PCP prophylaxis	HIV (*n* = 19)	Non-HIV (*n* = 49)
Immunocompromised status unknown to patient prior to the development of PCP	15 (78·9 %)	4 (8·2 %)
Taking immunosuppressive medication prior to the development of PCP	0 (0·0 %)	45 (91·8 %)
Ever on PCP prophylaxis prior to the development of PCP	0 (0·0 %)	21 (42·9 %)
Not on prescribed PCP prophylaxis at development of PCP	19 (100·0 %)	45 (91·8 %)

PCP, pneumocystis pneumonia; HIV, human immunodeficiency virus.

## Discussion

In the present study, we aimed to describe the current epidemiology of PCP in Germany. Therefore, we combined an analysis of secondary data on all inpatient hospital cases with a retrospective multi-center study over a concomitant period of five years. Our study produced three major findings: (1) the incidence of PCP has markedly increased in Germany between 2014 and 2019; (2) the spectrum of underlying immunosuppressive diseases has changed; and (3) PCP-related deaths are increasing significantly among non-HIV patients, who often do not receive chemoprophylaxis despite known immunosuppression.

Contrary to the findings of the relevant studies in the general population over the last 20 years, we found a recent increase in PCP incidence, with a peak of 2·6 per 100,000 persons in 2019. Previous longitudinal studies had observed a drastic decline in PCP worldwide, mainly due to the decreasing rates in HIV patients around the start of the new millennium.^1^^,^^2^^,^^6^, ^7^, ^8^ Subsequent epidemiologic investigations revealed a further decrease in PCP, followed by a steady low incidence rate: a decrease in the PCP incidence from 2·6 to 1·0 per 100,000 population was reported from 2001 to 2010 in a study based on the French national hospital database, which includes almost all inpatient cases.^9^ Another study using nationwide hospital data from Spain found that the PCP incidence was consistently about 2·0 per 100,000 population from 2008 to 2012.^11^ As for the incidence of PCP in other parts of the world, apart from rough estimates, there are currently no valid data at the population level.^5^ However, the number of people susceptible to developing PCP due to immunosuppression, other than HIV, is increasing worldwide: populations are aging; therefore, malignant diseases are increasing, and there have been significant advances in the treatment of autoimmune disorders.^14^^,^^15^^,^^17^ Based on these developments, we assume that the trend we observed of an increasing incidence of PCP in Germany is also applicable to other countries.

As for our second main finding, we found a distinct change in the spectrum of underlying diseases. The number of HIV patients with PCP continued to decrease. This development was quite surprising, because the prevalence of HIV had been increasing in Germany, according to the Robert-Koch-Institute, which is the leading federal research institution for public health in Germany.^18^ Our findings are, therefore, an indicator of adequate medical care and the consistent and successful use of PCP chemoprophylaxis for HIV patients. This assumption is supported by the fact that the absolute number of HIV-related deaths in Germany has declined yearly since 2014 (Figure S5).

The increase in the incidence of PCP in the general population that we observed is attributable entirely to an increase in the incidence in the HIV-negative risk groups. Additionally, the common course of illness among non-HIV patients was more severe than it was among HIV patients, in both our multi-center and nationwide studies. These observations are consistent with previous comparative center studies and a National Inpatient Sample Database study from the USA.^3^^,^^4^^,^^19^^,^^20^ Three subgroups of non-HIV patients were particularly affected by an increasing PCP incidence. First, the proportion of PCP patients with solid tumors has increased from 10·9% in 2014 to 14·5% in 2019. This trend was also evident in studies from Spain and the USA, but was masked in the overall analyses by the sharp declines in their HIV-related PCP cases.^11^^,^^19^ Second, the proportion of PCP patients with an isolated pulmonary disease as only risk factor significantly increased from 5·1 to 7·7%. Although frequent colonization of these patients by *Pneumocystis jirovecii* in the last century had been documented, these patients have only recently been identified as a group at considerable risk for PCP in epidemiological studies.^11^^,^^12^^,^^21^ We cannot specify the causes of this development, but our findings should encourage investigations that are more detailed. Third, the last of the three subgroups with a significant increase in PCP incidence consisted of patients to whom we could not assign an underlying immunosuppressive disease (16·8% of all identified PCP cases). A similar proportion was reported in the study conducted in Spain, which also investigated PCP cases using DRG data.^11^ Since PCP is an opportunistic illness that typically manifests only in immunocompromised individuals, these numbers seem surprising. In addition, only 2·9% of the PCP cases in our multi-center analysis did not have an underlying immunosuppressive disease. Therefore, the large proportion of PCP patients without apparent underlying diseases in our nationwide analysis may be due to the inability to detect diseases leading to immunosuppression among the DRG data in some cases. Due to the severe course of PCP in these patients and the fact that HIV infection is usually a prominent diagnosis, we conclude that these cases were correctly classified as non-HIV patients.

A general problem in researching PCP, which also affects our present study, is the uncertainty about how to confirm the diagnosis of PCP, as there is no uniformly accepted approach for this. Commonly, detection of *P. jirovecii* is considered easier in HIV patients, where in many cases induced sputum may be sufficient to detect the pathogen. In HIV-negative patients, on the other hand, invasive diagnostics for pathogen detection are supposed to be necessary more frequently. The introduction of the polymerase chain reaction for the detection of *P.jirovecii* in the 2000s has improved sensitivity, but it exacerbated another problem. Since the pathogen can colonize humans without causing disease, physicians are now confronted with the question as to the clinical significance of the pathogen detection and whether therapy should be initiated or not.^22^ The diagnostic uncertainty is underscored by the fact, that physicians have diagnosed PCP in some non-HIV patients included in our study even without definitive evidence of the pathogen. Consistent with the previous assumptions about the diagnostics of PCP, our data show that confirmation of a PCP diagnosis in non-HIV patients required more invasive diagnostics, pathogen detection was less successful, and therapy for these patients was started significantly later than in HIV patients. This delay could have posed a major problem for the affected individuals, since a delayed start of therapy implies a considerably worse prognosis.^4^^,^^23^

The most alarming major finding of our study was the significant increase in PCP-related deaths by 19·2% from 2014 to 2019. Taking into account a general increase in the annual deaths in Germany during the same time, a clear disproportionate increase in PCP fatalities of 10·2% could still be seen (Figure S6). Either way, our work is the first to show that an increase in PCP-related deaths in an industrialized country was entirely due to non-HIV causes. The increase was most striking in the group of patients with solid malignancies (+67·5%). We can rule out that this increase was part of a general increase in cancer-related deaths. Au contraire, the proportion of cancer-related deaths among all deaths in Germany actually decreased during the period under review (Figure S7).

We were not able to determine with certainty the reasons for the sharp increase in the PCP incidence and deaths among certain patients, as the methodology of our nationwide secondary data analysis did not allow us to examine individual cases, and the cohort of solid malignancy-related PCP cases in our multi-center study was too small to draw meaningful conclusions. What we do know for sure, however, is that the proper use of PCP prophylaxis could most likely prevent the vast majority of PCP cases and subsequent deaths.^24^ Unfortunately, we often fail in the adequate use of prophylaxis, which had already been observed in the past and was also evident from our own primary data.^25^ A major problem is the question of who needs prophylaxis, which must be clarified. Regarding this question, the implementation of PCP prophylaxis for non-HIV patients has been recommended in international guidelines and established in clinical practice for those with hematologic malignancies and those with organ transplantations.^26^, ^27^, ^28^ In contrast to all the other underlying diseases and conditions, these two groups of patients showed declines in the incidence of PCP and its related deaths. Therefore, in this study, we have provided evidence at the population level that PCP prophylaxis is effective in these patients. At the same time, our results provide an urgent reason to consider coordinated measures to establish PCP prophylaxis for other groups at risk, particularly high-risk patients with solid malignancies and multimorbid individuals.

Of course, it would be unrealistic to prescribe PCP prophylaxis to all patients in the identified risk groups. Further research is needed: the next reasonable step would be to evaluate the databases of large health insurers. In Germany, these companies have in-depth information on therapies and temporal relationships prior to the development of PCP, based on thousands of PCP cases. This information could be used to identify clusters of individuals with a particularly high risk of developing PCP. Ideally, randomized controlled trials on the efficacy of PCP prophylaxis, in particular for high-risk patient populations identified in this way would then be conducted, as has been done in the past for patients with hematological cancers and HIV, and kidney transplant recipients.^24^^,^^29^^,^^30^ Until then, it is incumbent upon treating physicians to be vigilant in managing each individual case and in prescribing PCP chemoprophylaxis outside of existing guidelines for potential high-risk patients.

## Conclusion

Since PCP is re-emerging, particularly in older patients with solid malignancies, morbidity rates and overall mortality will be higher than past rates. Physicians must be aware of this changing patient population and the urgent need for comprehensive guidelines for PCP prophylaxis for patients with solid malignancies and pulmonary and autoimmune disorders, as well as multimorbid patients. The reasons for the marked increase in PCP cases and deaths in patients with solid malignancies need further investigation.

## Limitations

The data of the German DRG statistics are the basis for the billing of hospital treatments in Germany, and are therefore audited by hospitals and health insurers. Nevertheless, the major limitation of using these data is that their accuracy cannot be verified on individual-case level. Their greatest advantage, however, is the enormous sample size that could never have been achieved in a single- or multi-center trial. On the other hand, the major limitation of the multi-center data is the small sample size and the resulting lack of representativeness for the nationwide situation. By comparing the primary patient data collected in our multi-center study with the nationwide secondary data analysis, we sought to provide more in-depth insight and uncover potential pitfalls in the secondary data. Apart from the overestimation of PCP patients without underlying diseases, the findings appear to be valid, and, to the best of our knowledge, we are not aware of any secondary data analysis of this size ever being directly paired with and supported by concomitant primary patient data. Finally, it should be noted that our data are most likely only representative of industrialized countries with similar demographics to those of Germany.

## Contributors

*Conceptualization:* Benedikt Kolbrink, Kevin Schulte

*Data curation:* Benedikt Kolbrink, Jubin Scheikholeslami-Sabzewari, Kevin Schulte, Christoph Borzikowsky

*Formal analysis:* Benedikt Kolbrink, Jubin Scheikholeslami-Sabzewari, Christoph Borzikowsky, Friedrich A. von Samson-Himmelstjerna, Andrew J. Ullmann, Kevin Schulte

*Investigation:* Benedikt Kolbrink, Jubin Scheikholeslami-Sabzewari, Kevin Schulte

*Project administration:* Benedikt Kolbrink, Kevin Schulte, Ulrich Kunzendorf

*Supervision:* Benedikt Kolbrink, Kevin Schulte, Ulrich Kunzendorf, Christoph Borzikowsky

*Visualization:* Benedikt Kolbrink, Jubin Scheikholeslami-Sabzewari

*Writing original draft:* Benedikt Kolbrink, Jubin Scheikholeslami-Sabzewari, Kevin Schulte

*Writing review and editing:* All authors

Benedikt Kolbrink, Jubin Scheikholeslami-Sabzewari and Kevin Schulte have directly accessed and verified the underlying data reported in the manuscript. All authors were responsible for the decision to submit the manuscript.

## Data availability statement

All the data used in the nationwide secondary data analysis are available for researchers upon request from the DRG statistics of the GFSO. The primary data from the retrospective multi-center study, which can be provided only in an anonymized and aggregated form due to data-protection regulations, are available upon reasonable request from the corresponding author.

## Declaration of interests

Kevin Schulte received honoraria for lectures from AstraZeneca, Novartis, Takeda Pharma and Vifor without relation to the study presented here. All other authors declare no competing interests.

## Appendix Supplementary materials

Image, application 1

Image, application 2

Image, application 3

## References

[1] Schwarcz L., Chen M.J., Vittinghoff E., Hsu L., Schwarcz S. (2013). Declining incidence of AIDS-defining opportunistic illnesses: results from 16 years of population-based AIDS surveillance. AIDS.

[2] Buchacz K., Baker R.K., Palella F.J. (2010). AIDS-defining opportunistic illnesses in US patients, 1994–2007: a cohort study. AIDS.

[3] Rego de Figueiredo I., Vieira Alves R., Drummond Borges D. (2019). Pneumocystosis pneumonia: a comparison study between HIV and non-HIV immunocompromised patients. Pulmonology.

[4] Roux A., Canet E., Valade S. (2014). Pneumocystis jirovecii pneumonia in patients with or without AIDS, France. Emerg Infect Dis.

[5] Bongomin F., Gago S., Oladele R.O., Denning DW. (2017). Global and multi-national prevalence of fungal diseases-estimate precision. J Fungi.

[6] Morris A., Lundgren J.D., Masur H. (2004). Current epidemiology of pneumocystis pneumonia. Emerg Infect Dis.

[7] Coelho L., Cardoso S.W., Amancio R.T. (2014). Trends in AIDS-defining opportunistic illnesses incidence over 25 years in Rio de Janeiro, Brazil. PLoS One.

[8] Wasserman S., Engel M.E., Griesel R., Mendelson M. (2016). Burden of pneumocystis pneumonia in HIV-infected adults in sub-Saharan Africa: a systematic review and meta-analysis. BMC Infect Dis.

[9] Bitar D., Lortholary O., Le Strat Y. (2014). Population-based analysis of invasive fungal infections, France, 2001–2010. Emerg Infect Dis.

[10] Wickramasekaran R.N., Jewell M.P., Sorvillo F., Kuo T. (2017). The changing trends and profile of pneumocystosis mortality in the United States, 1999–2014. Mycoses.

[11] Pereira-Diaz E., Moreno-Verdejo F., de la Horra C., Guerrero J.A., Calderon E.J., Medrano FJ. (2019). Changing trends in the epidemiology and risk factors of pneumocystis pneumonia in Spain. Front Public Health.

[12] Maini R., Henderson K.L., Sheridan E.A. (2013). Increasing pneumocystis pneumonia, England, UK, 2000–2010. Emerg Infect Dis.

[13] Gronseth S., Rogne T., Hannula R., Asvold B.O., Afset J.E., Damas JK. (2021). Epidemiological and clinical characteristics of immunocompromised patients infected with pneumocystis jirovecii in a twelve-year retrospective study from Norway. BMC Infect Dis.

[14] Harpaz R., Dahl R.M., Dooling KL. (2016). Prevalence of Immunosuppression among US Adults, 2013. JAMA.

[15] Yancik R., Ries L.A. (2004). Cancer in older persons: an international issue in an aging world. Semin Oncol.

[16] The database of the German federal statistical office. 2021. https://www-genesis.destatis.de/genesis/online. Accessed 2 November 2021.

[17] Reinhardt U.E., Hussey P.S., Anderson G.F. (2002). Cross-national comparisons of health systems using OECD data, 1999. Health Aff.

[18] an der Heiden M., Marcus U., Kollan C., Schmidt D., Gunsenheimer-Bartmeyer B., Bremer V. (2020). Schätzung der Zahl der HIV-Neuinfektionen und der Gesamtzahl von Menschen mit HIV in Deutschland, Stand Ende 2019. Epid Bull.

[19] Kanj A., Samhouri B., Abdallah N., Chehab O., Baqir M. (2021). Host factors and outcomes in hospitalizations for pneumocystis jirovecii pneumonia in the United States. Mayo Clin Proc.

[20] Nuesch R., Bellini C., Zimmerli W. (1999). Pneumocystis carinii pneumonia in human immunodeficiency virus (HIV)-positive and HIV-negative immunocompromised patients. Clin Infect Dis.

[21] Calderon E.J., Regordan C., Medrano F.J., Ollero M., Varela J.M. (1996). Pneumocystis carinii infection in patients with chronic bronchial disease. Lancet.

[22] Cillóniz C., Dominedò C., Álvarez-Martínez M.J. (2019). Pneumocystis pneumonia in the twenty-first century: HIV-infected versus HIV-uninfected patients. Expert Rev Anti Infect Ther.

[23] Li M.C., Lee N.Y., Lee C.C., Lee H.C., Chang C.M., Ko W.C. (2014). Pneumocystis jiroveci pneumonia in immunocompromised patients: delayed diagnosis and poor outcomes in non-HIV-infected individuals. J Microbiol Immunol Infect.

[24] Stern A., Green H., Paul M., Vidal L., Leibovici L. (2014). Prophylaxis for Pneumocystis pneumonia (PCP) in non-HIV immunocompromised patients. Cochr Database Syst Rev.

[25] Dunbar A., Schauwvlieghe A., Algoe S. (2020). Epidemiology of pneumocystis jirovecii pneumonia and (Non-)use of prophylaxis. Front Cell Infect Microbiol.

[26] Maertens J., Cesaro S., Maschmeyer G. (2016). ECIL guidelines for preventing Pneumocystis jirovecii pneumonia in patients with haematological malignancies and stem cell transplant recipients. J Antimicrob Chemother.

[27] Kasiske B.L., Zeier M.G., Chapman J.R. (2010). KDIGO clinical practice guideline for the care of kidney transplant recipients: a summary. Kidney Int.

[28] Classen A.Y., Henze L., von Lilienfeld-Toal M. (2021). Primary prophylaxis of bacterial infections and pneumocystis jirovecii pneumonia in patients with hematologic malignancies and solid tumors: 2020 updated guidelines of the Infectious diseases working party of the German society of hematology and medical oncology (AGIHO/DGHO). Ann Hematol.

[29] Fischl M.A., Dickinson G.M., La Voie L. (1988). Safety and efficacy of sulfamethoxazole and trimethoprim chemoprophylaxis for pneumocystis carinii pneumonia in AIDS. JAMA.

[30] Schneider M.M., Hoepelman A.I., Eeftinck Schattenkerk J.K. (1992). A controlled trial of aerosolized pentamidine or trimethoprim-sulfamethoxazole as primary prophylaxis against pneumocystis carinii pneumonia in patients with human immunodeficiency virus infection. The Dutch AIDS treatment group. N Engl J Med.

