# Supplementary material for: Evolving epidemiology of pneumocystis pneumonia: Findings from a longitudinal population-based study and a retrospective multi-center study in Germany
Source: Lancet Reg Health Eur. 2022 May 15;18:100400. doi: 10.1016/j.lanepe.2022.100400 (PMC9257643; doi:10.1016/j.lanepe.2022.100400)
Supplement: Supplementary file 1 [file mmc1.pdf]

Supplementary Appendix to ***Evolving Epidemiology of Pneumocystis Pneumonia: Findings from a Longitudinal Population-Based Study and a Retrospective Multi-Center Study in Germany***

Table of content

Section	Page
**Supplementary Figures**	
Supplementary Figure 1	2
Supplementary Figure 2	3
Supplementary Figure 3	3
Supplementary Figure 4	4
Supplementary Figure 5	5
Supplementary Figure 6	6
Supplementary Figure 7	7
**Supplementary Tables**	
Supplementary Table 1	8
Supplementary Table 2	9
Supplementary Table 3	12
Supplementary Table 4	13
Supplementary Table 5	14
Supplementary Table 6	15
Supplementary Table 7	16
Supplementary Table 8	16
Supplementary Table 9	17
**Supplementary References**	18

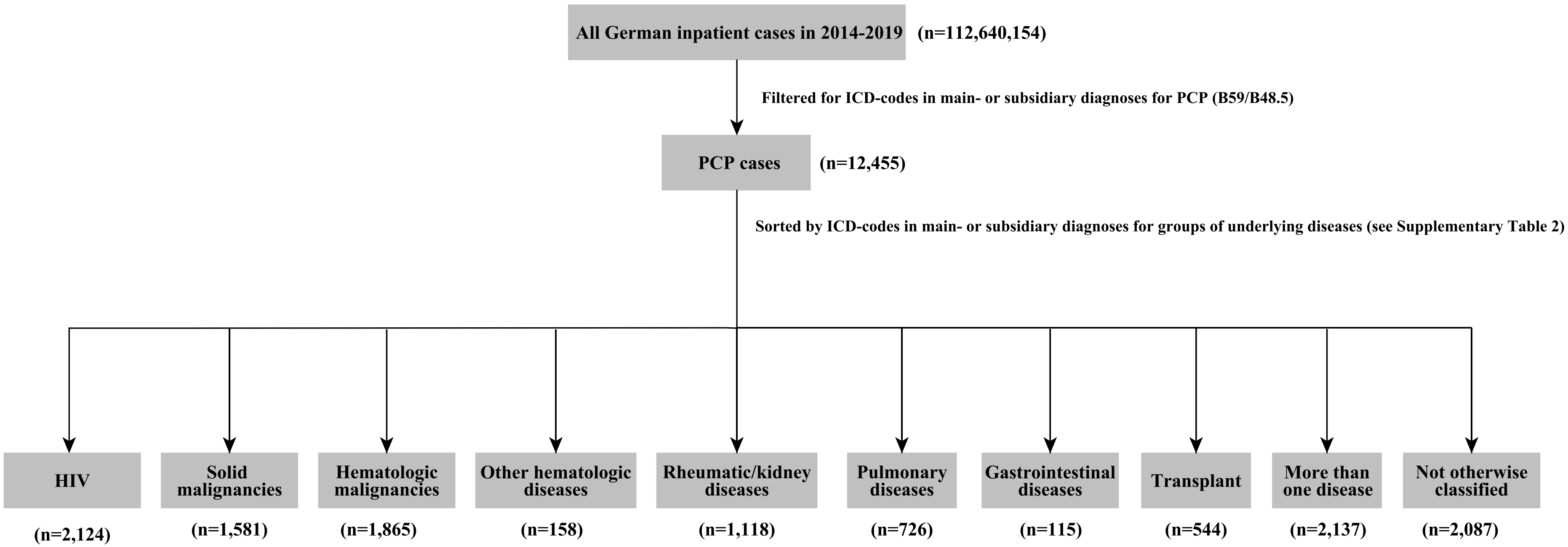

Supplementary Figure 1. Analytic algorithm for identification of PCP cases and risk factors in the nationwide secondary data

For technical reasons, this algorithm had to be applied to each year under consideration separately. Indicated patient numbers represent the total sum of all patients from 2014 to 2019.

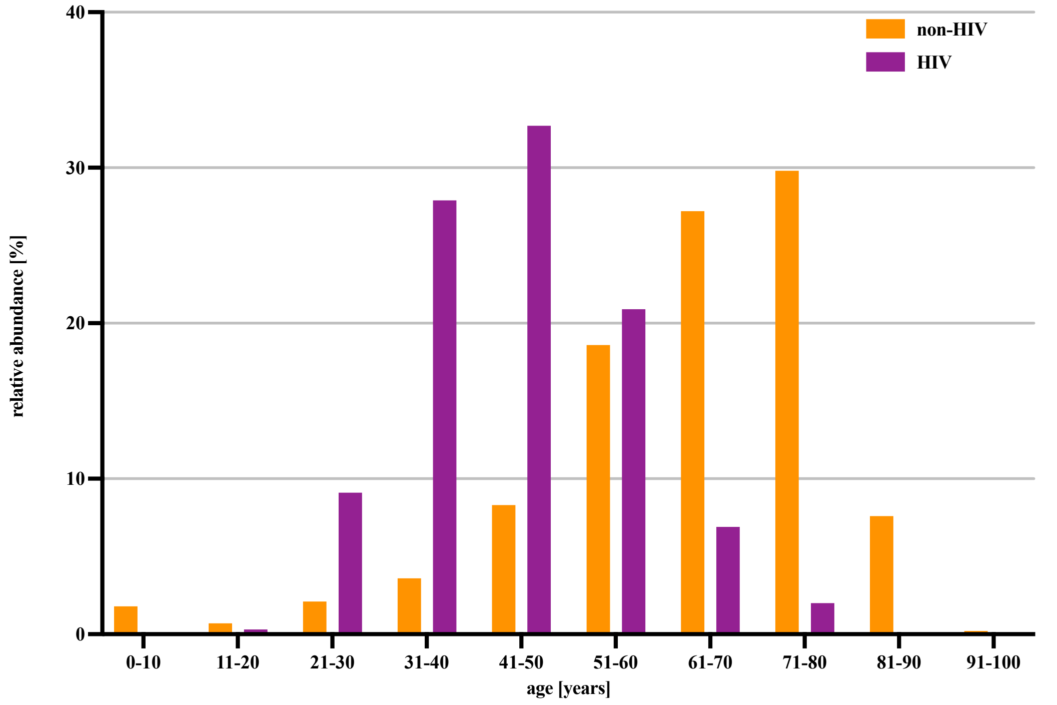

Supplementary Figure 2. Age distribution of HIV and non-HIV PCP patients in Germany

Relative abundance of HIV and non-HIV PCP patients across age groups is shown. PCP patients were identified from the DRG statistics of the German Federal Statistical Office from 2014 to 2019. For both groups, the percentages sum up to 100 each. This graph does not reflect the difference in absolute numbers between the HIV and non-HIV group (2,124 HIV vs. 10,331 non-HIV patients, respectively).

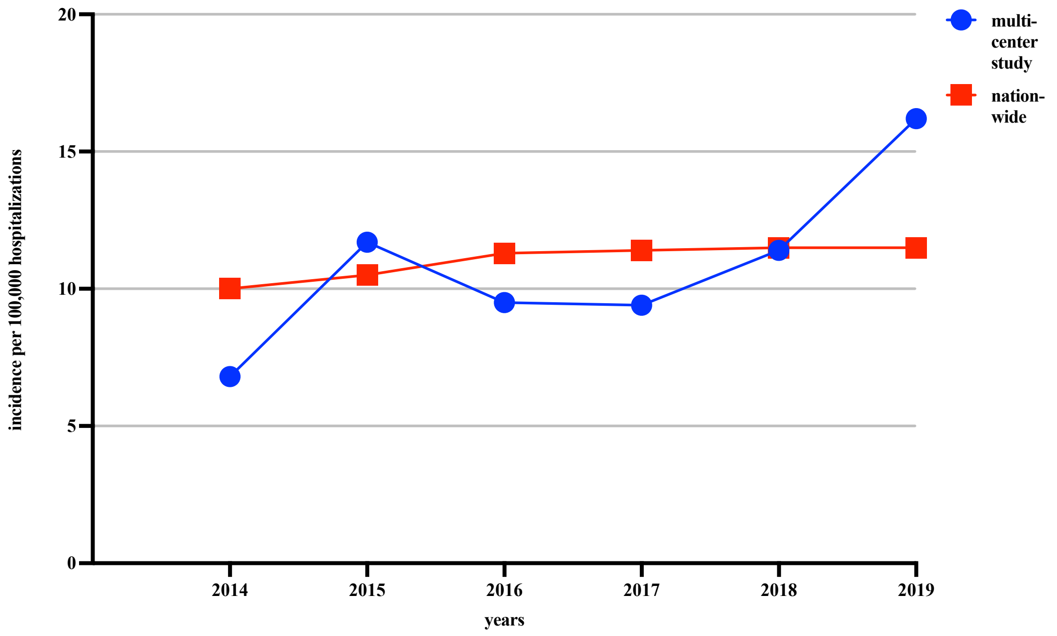

Supplementary Figure 3. PCP incidence multi-center study vs. nationwide

The graph shows the incidence of PCP cases per 100,000 hospitalizations in our multi-center study (blue) and the secondary data analysis of all German inpatient cases (red) from 2014 to 2019. Averaged over the entire observation period, the incidence was 10·5 in the multi-center study and 11·1 per 100,000 hospitalizations in the nationwide analysis.

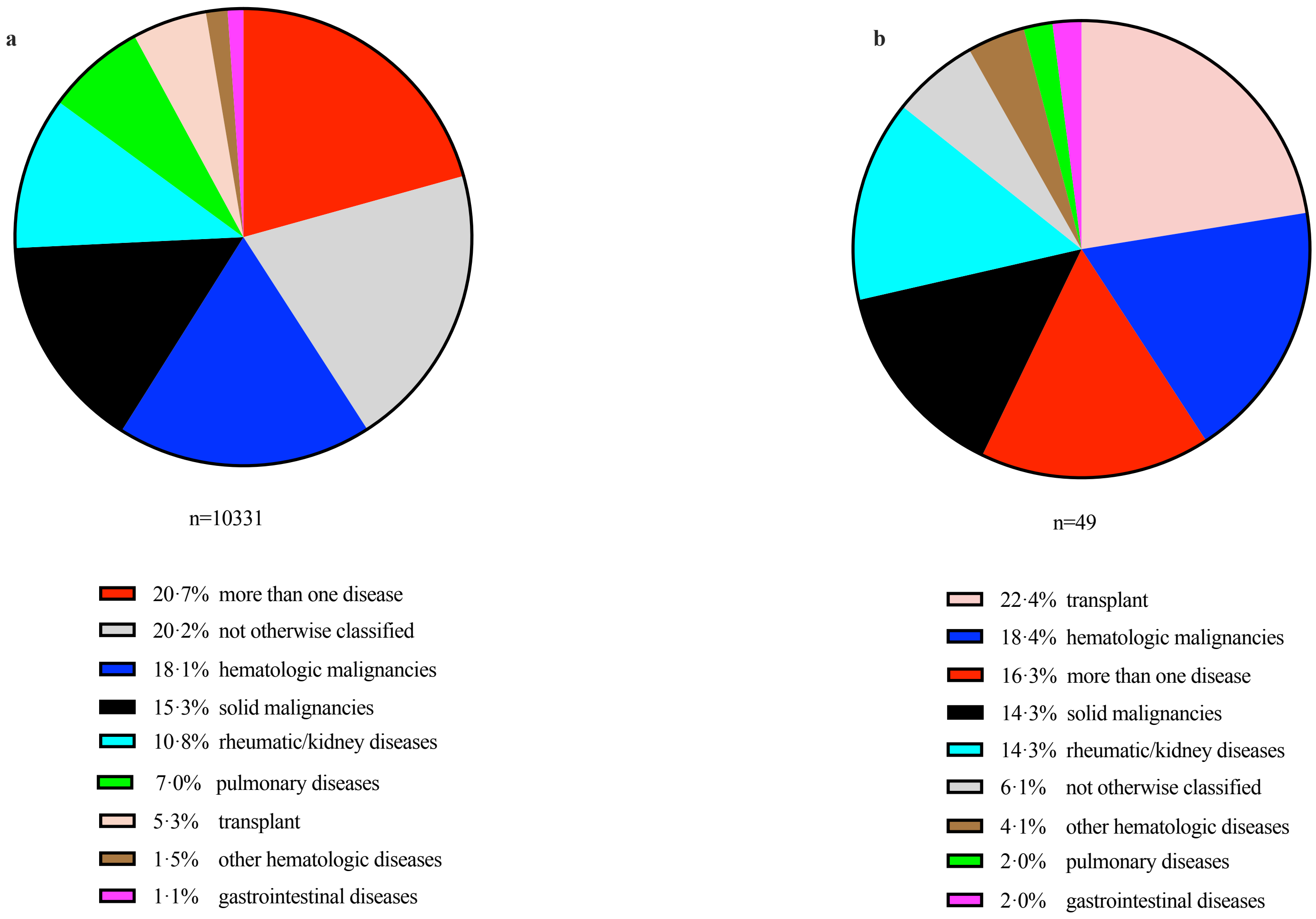

Supplementary Figure 4. Underlying diseases other than HIV of PCP in the multi-center study vs. nationwide

Pie charts depict the distribution of underlying diseases and conditions for the development of PCP among the non-HIV group in the nationwide secondary data analysis (a) and the concomitant multi-center study (b). Noteworthy is an overrepresentation of the group “transplant” in the multi-center study. This is most likely due to the fact that the clinics under investigation here are transplantation centers that treat a significantly larger number of organ transplant recipients than most German hospitals. Reasons for the clear predominance of the "not otherwise classified" group in the nationwide secondary data analysis are elaborated on in the "discussion" section.

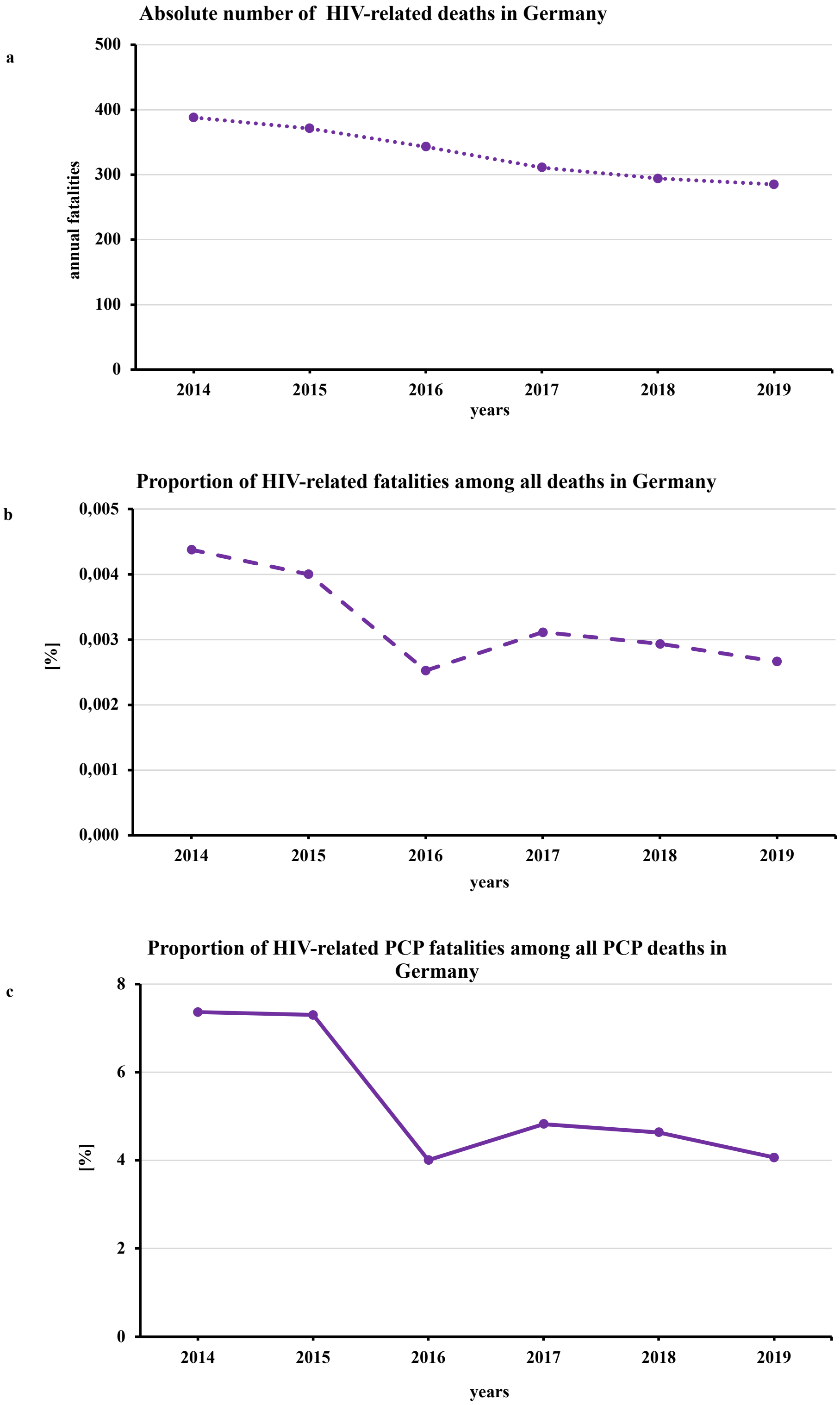

Supplementary Figure 5. HIV related deaths in Germany 2014 to 2019

(a) The number of HIV-related fatalities in Germany decreased by 26·5% from 2014 to 2019. This was accompanied by a decrease in the proportion of HIV-related deaths among all deaths in Germany (b).[1] The decline in absolute and relative death rates was also reflected in the proportion of HIV-related PCP deaths among all PCP-related deaths (c).

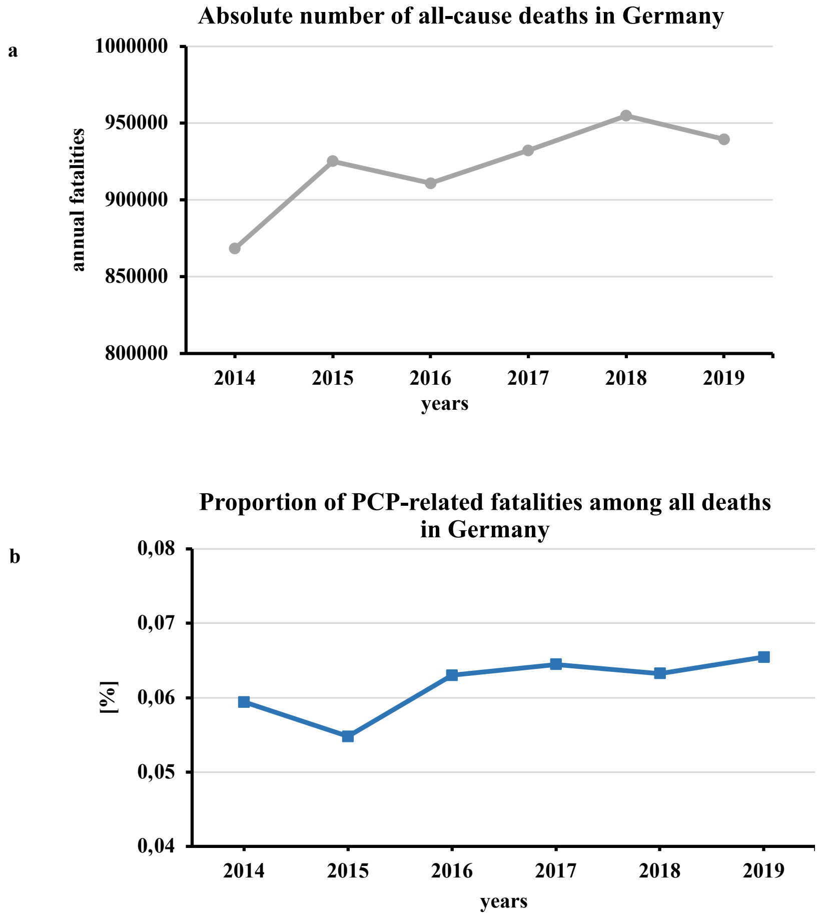

Supplementary Figure 6. Proportion of PCP-related deaths in all fatalities in Germany 2014 to 2019

(a) The number of all-cause deaths in Germany increased from 868,356 to 939,520 between 2014 and 2019.[1]
(b) The proportion of PCP-related deaths herein nevertheless increased by 9·2% (from 59 in 100,000 deaths to 65 in 100,000 deaths).

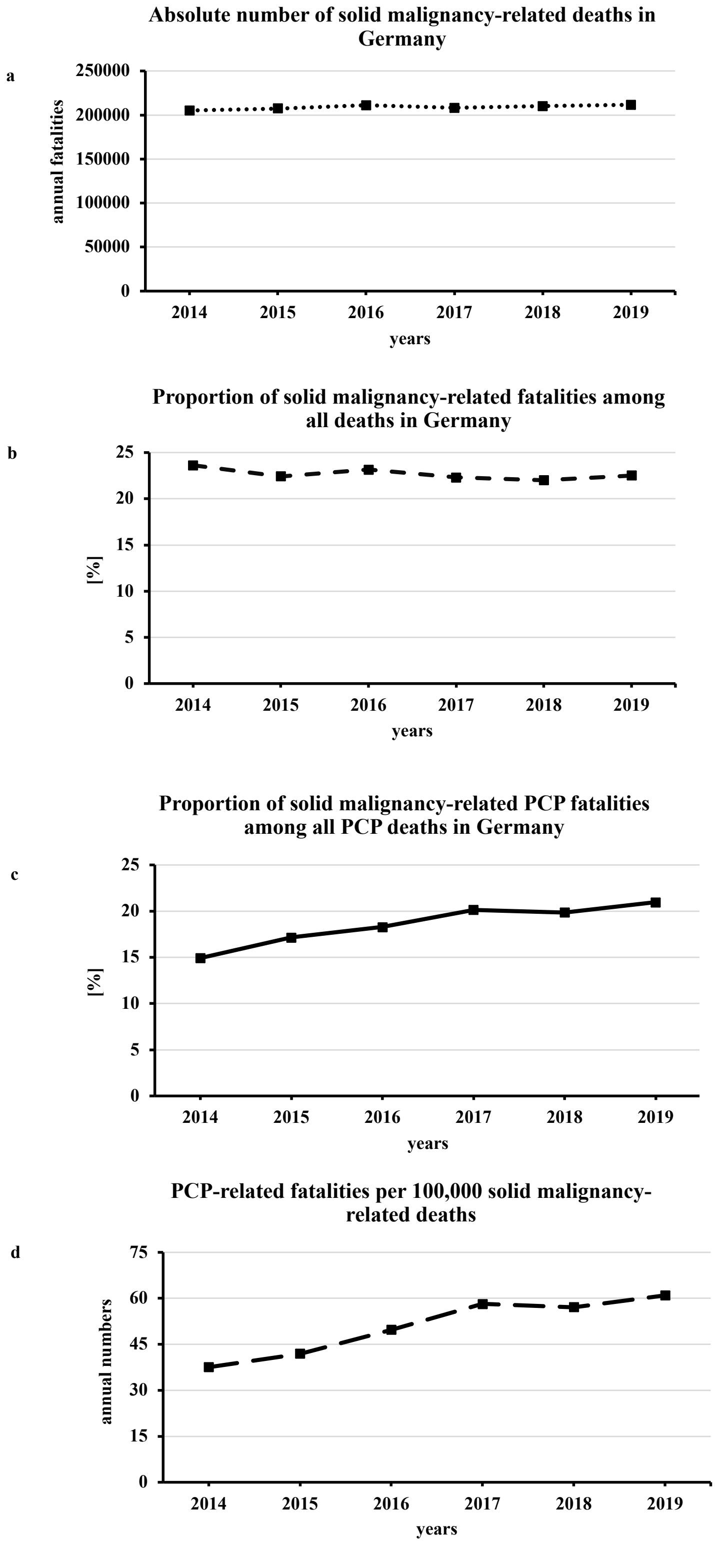

Supplementary Figure 7. Development of solid malignancy-related deaths and proportion of PCP-related deaths therein from 2014 to 2019

(a) The number of deaths caused by solid malignancies in Germany increased slightly from 2014 to 2019 (205,149 to 211,601), (b) and the proportion of these among all deaths decreased slightly (23·6% to 22·5%).[1] (c) At the same time, the proportion of solid malignancy-related PCP fatalities among all PCP-related deaths in Germany increased sharply (14·9% to 23·0%), (d) as well as the number of PCP-related fatalities per 100,000 solid malignancy- related deaths (37·5 to 61·0 per 100,000 deaths).

Supplementary Table 1. Diagnostic-, inpatient course-, and organ dysfunction codes

**German Operation and Procedure Classification System key codes for diagnostic and therapeutic procedures**	
Tracheobronchoscopy and<br>bronchial lavage	1-620<br>1-843
Thorax CT	3-202<br>3-222
Thorax MRI	3-809<br>3-822
Intensive care treatment	8-980<br>8-98d<br>8-98f
Mechanical ventilation	8-70<br>8-71<br>8-72
Renal replacement therapy	8-853<br>8-854<br>8-855<br>8-856<br>8-857
Palliative care treatment	8-982<br>8-98e<br>8-98h
Cardiopulmonary resuscitation	8-77
**ICD-10 codes for organ dysfunctions**	
Cardiovascular	I95.9: Hypotension, unspecified<br>R57.9: Shock, unspecified<br>R57.8: Other shock
Respiratory	J96: Respiratory failure, not elsewhere classified<br>J96.9: Respiratory failure, unspecified<br>J80: Acute respiratory distress syndrome<br>J98.4: Other disorders of lung<br>R06.0: Dyspnea<br>R06.8: Other abnormalities of breathing
Central nervous system	F05: Delirium<br>G93.1: Anoxic brain damage<br>G93.4: Encephalopathy<br>R40: Somnolence, stupor and coma
Renal	N17: Acute kidney failure<br>N19: Unspecified kidney failure
Metabolic	E87.2: Acidosis
Hematologic	D65: Disseminated intravascular coagulation<br>D68.8: Other specified coagulation defects<br>D68.9: Coagulation defect, unspecified<br>D69.5: Secondary thrombocytopenia<br>D69.6: Thrombocytopenia, unspecified
Hepatic	K72.0: Acute and subacute hepatic failure<br>K76.2: Central hemorrhagic necrosis of liver<br>K72.7-!: Hepatic encephalopathy and hepatic coma<br>K76.3: Infarction of liver
Multi-organ dysfunction	R65.1!: SIRS due to infection with organ dysfunction<br>R57.2: Septic shock

CT, computed tomography; MRI, magnetic resonance imaging; SIRS, systemic inflammatory response syndrome

Supplementary Table 2. ICD-10 codes for immunosuppressive conditions

Underlying diseases	Corresponding ICD-10 codes
HIV	B20.- HIV disease resulting in infectious and parasitic diseases<br>B21.- HIV disease resulting in malignant neoplasms<br>B22.- HIV disease resulting in other specified diseases<br>B23.- HIV disease resulting in other conditions<br>B24.- Unspecified HIV disease
Solid malignancies	C00.- Malignant neoplasm of lip<br>C01.- Malignant neoplasm of base of tongue<br>C02.- Malignant neoplasm of other and unspecified parts of tongue<br>C03.- Malignant neoplasm of gum<br>C04.- Malignant neoplasm of floor of mouth<br>C05.- Malignant neoplasm of palate<br>C06.- Malignant neoplasm of other and unspecified parts of mouth<br>C07.- Malignant neoplasm of parotid gland<br>C08.- Malignant neoplasm of other and unspecified major salivary glands<br>C09.- Malignant neoplasm of tonsil<br>C10.- Malignant neoplasm of oropharynx<br>C11.- Malignant neoplasm of nasopharynx<br>C12.- Malignant neoplasm of piriform sinus<br>C13.- Malignant neoplasm of hypopharynx<br>C14.- Malignant neoplasm of other and ill-defined sites in the lip, oral cavity and pharynx<br>C15.- Malignant neoplasm of esophagus<br>C16.- Malignant neoplasm of stomach<br>C17.- Malignant neoplasm of small intestine<br>C18.- Malignant neoplasm of colon<br>C19.- Malignant neoplasm of rectosigmoid junction<br>C20.- Malignant neoplasm of rectum<br>C21.- Malignant neoplasm of anus and anal canal<br>C22.- Malignant neoplasm of liver and intrahepatic bile ducts<br>C23.- Malignant neoplasm of gallbladder<br>C24.- Malignant neoplasm of other and unspecified parts of biliary tract<br>C25.- Malignant neoplasm of pancreas<br>C26.- Malignant neoplasm of other and ill-defined digestive organs<br>C30.- Malignant neoplasm of nasal cavity and middle ear<br>C31.- Malignant neoplasm of accessory sinuses<br>C32.- Malignant neoplasm of larynx<br>C33.- Malignant neoplasm of trachea<br>C34.- Malignant neoplasm of bronchus and lung<br>C37.- Malignant neoplasm of thymus<br>C38.- Malignant neoplasm of heart, mediastinum and pleura<br>C39.- Malignant neoplasm of other and ill-defined sites in the respiratory system and intrathoracic organs<br>C40.- Malignant neoplasm of bone and articular cartilage of limbs<br>C41.- Malignant neoplasm of bone and articular cartilage of other and unspecified sites<br>C43.- Malignant neoplasm of skin<br>C44.- Other malignant neoplasms of skin

	C45.- Mesothelioma<br>C46.- Kaposi’s sarcoma<br>C47.- Malignant neoplasm of peripheral nerves and automatic nervous system<br>C48.- Malignant neoplasm of retroperitoneum and peritoneum<br>C49.- Malignant neoplasm of other connective and soft tissue<br>C50.- Malignant neoplasm of breast<br>C51.- Malignant neoplasm of vulva<br>C52.- Malignant neoplasm of vagina<br>C53.- Malignant neoplasm of cervix uteri<br>C54.- Malignant neoplasm of corpus uteri<br>C55.- Malignant neoplasm of uterus, part unspecified<br>C56.- Malignant neoplasm of ovary<br>C57.- Malignant neoplasm of other and unspecified female genital organs<br>C58.- Malignant neoplasm of placenta<br>C60.- Malignant neoplasm of penis<br>C61.- Malignant neoplasm of prostate<br>C62.- Malignant neoplasm of testis<br>C63.- Malignant neoplasm of other and unspecified male genital organs<br>C64.- Malignant neoplasm of kidney, except renal pelvis<br>C65.- Malignant neoplasm of renal pelvis<br>C66.- Malignant neoplasm of ureter<br>C67.- Malignant neoplasm of bladder<br>C68.- Malignant neoplasm of other and unspecified urinary organs<br>C69.- Malignant neoplasm of eye and adnexa<br>C70.- Malignant neoplasm of meninges<br>C71.- Malignant neoplasm of brain<br>C72.- Malignant neoplasm of spinal cord, cranial nerves and other parts of central nervous system<br>C73.- Malignant neoplasm of thyroid gland<br>C74.- Malignant neoplasm of adrenal gland<br>C75.- Malignant neoplasm of other endocrine glands and related structures
Hematologic malignancies	C81.- Hodgkin lymphoma<br>C82.- Follicular lymphoma<br>C83.- Non-follicular lymphoma<br>C84.- Mature T/NK-cell lymphomas<br>C85.- Other and unspecified types of non-Hodgkin lymphoma<br>C86.- Other specified types of T/NK-cell lymphoma<br>C88.- Malignant immunoproliferative diseases<br>C90.- Multiple myeloma and malignant plasma cell neoplasms<br>C91.- Lymphoid leukemia<br>C92.- Myeloid leukemia<br>C93.- Monocytic leukemia<br>C94.- Other leukemias of specified cell type<br>C95.- Leukemia of unspecified cell type<br>C96.- Other and unspecified malignant neoplasms of lymphoid, hematopoietic and related tissue<br>C97.- Malignant neoplasms of independent (primary) multiple sites<br>D46.- Myelodysplastic syndromes

	D47.- Other neoplasms of uncertain or unknown behaviour of lymphoid, hematopoietic and related tissue<br>Z94.81 Condition after hematopoietic stem cell transplantation with current immunosuppression
Other hematologic diseases	D59.- Acquired hemolytic anemia<br>D60.- Acquired pure red cell aplasia<br>D61.- Other aplastic anemias<br>D76.- Other specified diseases with participation of lymphoreticular and reticulohistiocytic tissue<br>D80.- Immunodeficiency with predominantly antibody defects<br>D81.- Combined immunodeficiencies<br>D82.- Immunodeficiency associated with other major defects<br>D83.- Common variable immunodeficiency<br>D84.- Other immunodeficiencies
Rheumatic/kidney diseases	D86.- Sarcoidosis<br>M30.- Polyarteritis nodosa and related conditions<br>M31.- Other necrotizing vasculopathies<br>M32.- Systemic lupus erythematosus<br>M33.- Dermatopolymyositis<br>M34.- Systemic sclerosis<br>M35.- Other systemic involvement of connective tissue<br>M05.- Seropositive rheumatoid arthritis<br>M06.- Other rheumatoid arthritis<br>M07.-* Psoriatic and enterohepatic arthropathies<br>M08.- Juvenile arthritis<br>M09.-* Juvenile arthritis in diseases classified elsewhere<br>M12.- Other specific arthropathies<br>M13.- Other arthritis<br>M14.-* Arthropathies in other diseases classified elsewhere<br>M45.- Ankylosing spondylitis<br>N00.- Acute nephritic syndrome<br>N01.- Rapidly progressive nephritic syndrome<br>N03.- Chronic nephritic syndrome<br>N04.- Nephrotic syndrome<br>L10.- Pemphigus<br>L12.- Pemphigoid<br>L13.- Other bullous disorders<br>L14.-* Bullous disorders in diseases classified elsewhere
Pulmonary diseases	J43.- Emphysema<br>J44.- Other chronic obstructive pulmonary disease<br>J45.- Asthma<br>J60.- Coalworker's pneumoconiosis<br>J61.- Pneumoconiosis due to asbestos and other mineral fibers<br>J62.- Pneumoconiosis due to dust containing silica<br>J63.- Pneumoconiosis due to other organic dusts<br>J64.- Unspecified pneumoconiosis<br>J65.- Pneumoconiosis associated with tuberculosis<br>J66.- Airway disease due to specific organic dust<br>J67.- Hypersensitivity pneumonitis due to organic dust<br>J68.- Respiratory conditions due to inhalation of chemicals, gases, fumes and vapors<br>J69.- Pneumonitis due to solids and liquids<br>J70.- Respiratory conditions due to other external agents

	E84.- Cystic fibrosis
Gastrointestinal diseases	K50.- Crohn disease (regional enteritis)<br>K51.- Ulcerative colitis<br>K75.4.- Autoimmune hepatitis
Transplant	Z94.0.- Kidney transplant status<br>Z94.1.- Heart transplant status<br>Z94.2.- Lung transplant status<br>Z94.3.- Heart and lungs transplant status<br>Z94.4.- Liver transplant status

HIV, human immunodeficiency virus; NK, natural killer cell

Supplementary Table 3. Changes in PCP cases and deaths from 2014 to 2019

Year	2014	2019	p-value (Chi-square test)
PCP cases	1,857	2,172	<0·0001
PCP deaths	516	615	0·0111

PCP, pneumocystis pneumonia

For the test of statistical significance, the population data provided by the German Federal Statistical Office as of December, 31 of the respective years were used (81,197,537 people in 2014 and 83,166,711 people in 2019).[1]

Supplementary Table 4. Changes in PCP cases among different risk groups from 2014 to 2019

Underlying diseases	Case number 2014	Case number 2019	Relative change (%)	p-value (Fisher's exact test)
HIV	346	331	-4·3%	0·0046
Solid malignancies	203	316	55·7%	0·0007
Hematologic malignancies	326	270	-17·2%	<0·0001
Other hematologic diseases	28	29	3·6%	0·6890
Rheumatic/kidney diseases	170	201	18·2%	0·9564
Pulmonary diseases	95	167	75·8%	0·0010
Gastrointestinal diseases	11	19	72·7%	0·3596
Transplant	76	67	-11·8%	0·0880
More than one disease	337	373	10·7%	0·4308
Not otherwise classified	265	399	50·6%	0·0005

PCP, pneumocystis pneumonia; HIV, human immunodeficiency virus

Supplementary Table 5. Changes in PCP-related deaths among different risk groups from 2014 to 2019

Underlying diseases	PCP-related deaths 2014	PCP-related deaths 2019	Relative change (%)	p-value (Fisher's exact test)
HIV	38	25	-34·2%	0·0188
Solid malignancies	77	129	67·5%	0·0086
Hematologic malignancies	117	88	-24·8%	0·0003
Other hematologic diseases	7	6	-14·3%	0·5860
Rheumatic/kidney diseases	41	58	41·5%	0·3996
Pulmonary diseases	26	36	38·5%	0·6010
Gastrointestinal diseases	4	5	25%	>0·9999
Transplant	18	17	-5·6%	0·4958
More than one disease	95	115	21·1%	0·9389
Not otherwise classified	93	136	46·2%	0·1021

PCP, pneumocystis pneumonia; HIV, human immunodeficiency virus

Supplementary Table 6. Diagnostic procedures performed on PCP patients for each respective year (nationwide)

Year	Procedure	HIV	Non-HIV	p-value HIV vs. non-HIV (Chi-square test)
2014	Thorax CT/MRI	202/346 (58·4 %)	1,091/1,511 (72·2 %)	<0·0001
	Bronchoscopy	239/346 (69·1 %)	1,089/1,511 (72·1 %)	0·2653
	Thorax CT/MRI and bronchoscopy	157/346 (45·4 %)	846/1,511 (56·0 %)	0·0004
2015	Thorax CT/MRI	241/399 (60·4 %)	1,143/1,568 (72·9 %)	<0·0001
	Bronchoscopy	267/399 (66·9 %)	1,129/1,568 (72·0 %)	0·0457
	Thorax CT/MRI and bronchoscopy	189/399 (47·4 %)	865/1,568 (55·2 %)	0·0053
2016	Thorax CT/MRI	215/356 (60·4 %)	1,308/1,785 (73·3 %)	<0·0001
	Bronchoscopy	231/356 (64·9 %)	1,281/1,785 (71·8 %)	0·0093
	Thorax CT/MRI and bronchoscopy	159/356 (44·7 %)	1,010/1,785 (56·6 %)	<0·0001
2017	Thorax CT/MRI	223/351 (63·5 %)	1,332/1,805 (73·8 %)	<0·0001
	Bronchoscopy	236/351 (67·2 %)	1,294/1,805 (71·7 %)	0·0926
	Thorax CT/MRI and bronchoscopy	170/351 (48·4 %)	1,043/1,805 (57·8 %)	0·0012
2018	Thorax CT/MRI	205/341 (60·1 %)	1,369/1,821 (75·2 %)	<0·0001
	Bronchoscopy	238/341 (69·8 %)	1,316/1,821 (72·3 %)	0·3512
	Thorax CT/MRI and bronchoscopy	173/341 (50·7 %)	1,057/1,821 (58·0 %)	0·0123
2019	Thorax CT/MRI	221/331 (66·8 %)	1,382/1,841 (75·1 %)	0·0016
	Bronchoscopy	229/331 (69·2 %)	1,345/1,841 (73·1 %)	0·1463
	Thorax CT/MRI and bronchoscopy	165/331 (49·8 %)	1,085/1,841 (58·9 %)	0·0021

CT, computed tomography; MRI, magnetic resonance imaging; HIV, human immunodeficiency virus

Supplementary Table 7. Diagnostic procedures by underlying diseases (nationwide)

Procedure	HIV (n=2124)	Solid malignancies (n=1581)	Hematologic malignancies (n=1865)	Other hematologic diseases (n=158)	Rheumatic/kidney diseases (n=1118)	Pulmonary diseases (n=726)	Gastrointestinal diseases (n=115)	Transplant (n=544)	More than one disease (n=2137)	Not otherwise classified (n=2087)
Thorax CT/MRI	1,307 (61·5%)	1,191 (75·3%)	1,489 (79·8%)	92 (58·2%)	797 (71·3%)	482 (66·4%)	84 (73·0%)	412 (75·7%)	1,676 (78·4%)	1,402 (67·2%)
Bronchoscopy	1,440 (67·8%)	1,096 (69·3%)	1,296 (69·5%)	103 (65·2%)	842 (75·3%)	555 (76·4%)	93 (80·9%)	402 (73·9%)	1,598 (74·8%)	1,469 (70·4%)
Thorax CT/MRI and bronchoscopy	1,013 (47·7%)	877 (55·5%)	1,080 (57·9%)	73 (46·2%)	661 (59·1%)	382 (52·6%)	71 (61·7%)	333 (61·2%)	1,325 (62·0%)	1,104 (52·9%)

CT, computed tomography; MRI, magnetic resonance imaging; HIV, human immunodeficiency virus

Supplementary Table 8. Inpatient course and complications by underlying diseases (nationwide)

Event	HIV (n=2,124)	Solid malignancies (n=1,581)	Hematologic malignancies (n=1,865)	Other hematologic diseases (n=158)	Rheumatic/kidney diseases (n=1,118)	Pulmonary diseases (n=726)	Gastrointestinal diseases (n=115)	Transplant (n=544)	More than one disease (n=2,137)	Not otherwise classified (n=2,087)
Death	180 (8·5 %)	639 (40·4 %)	616 (33·0 %)	31 (19·6 %)	309 (27·6 %)	191 (26·3 %)	30 (26·1 %)	102 (18·8 %)	638 (29·9 %)	681 (32·6 %)
ICU care	705 (33·2 %)	693 (43·8 %)	802 (43·0 %)	86 (54·4 %)	585 (52·3 %)	318 (43·8 %)	59 (51·3 %)	258 (47·4 %)	960 (44·9 %)	1,028 (49·3 %)
Mechanical ventilation	574 (27·0 %)	677 (42·8 %)	769 (41·2 %)	75 (47·5 %)	546 (48·8 %)	315 (43·4 %)	60 (52·2 %)	220 (40·4 %)	908 (42·5 %)	998 (47·8 %)
Renal replacement therapy	106 (5·0 %)	100 (6·3 %)	252 (13·5 %)	20 (12·7 %)	195 (17·4 %)	89 (12·3 %)	17 (14·8 %)	220 (40·4 %)	262 (12·3 %)	402 (19·3 %)
Multiple organ dysfunction	422 (19·9 %)	590 (37·3 %)	910 (48·8 %)	76 (48·1 %)	496 (44·4 %)	327 (45·0 %)	60 (52·2 %)	243 (44·7 %)	955 (44·7 %)	944 (45·2 %)
Palliative care	0 (0·0 %)	205 (13·0 %)	62 (3·3 %)	0 (0·0 %)	1 (0·1 %)	7 (1·0 %)	1 (0·9 %)	1 (0·2 %)	94 (4·4 %)	21 (1·0 %)
Cardiac arrest/ Resuscitation	37 (1·7 %)	40 (2·5 %)	88 (4·7 %)	6 (3·8 %)	64 (5·7 %)	43 (5.9 %)	1 (0·9 %)	19 (3·5 %)	75 (3·5 %)	111 (5·3 %)

HIV, human immunodeficiency virus; ICU, intensive care unit

Supplementary Table 9. Inpatient course and complications by underlying diseases (multi-center study)

	**HIV (n=19)**	**Solid malignancies (n=7)**	**Hematologic malignancies (n=9)**	**Other hematologic diseases (n=2)**	**Rheumatic/kidney diseases (n=7)**	**Pulmonary diseases (n=1)**	**Gastrointestinal diseases (n=1)**	**Transplant (n=11)**	**More than one disease (n=8)**	**Not otherwise classified (n=3)**
Deaths	1 (5·3 %)	3 (42·9 %)	2 (22·2 %)	1 (50 %)	1 (14·3 %)	1 (100·0 %)	1 (100·0 %)	0 (0·0 %)	4 (50 %)	1 (33·3 %)
ICU	5 (26·3 %)	1 (14·3 %)	3 (33·3 %)	1 (50 %)	4 (57·1 %)	1 (100·0 %)	1 (100·0 %)	4 (36·4 %)	3 (37·5 %)	2 (66·7 %)
Mechanical ventilation	8 (42·1 %)	5 (71·4 %)	5 (55·6 %)	1 (50 %)	5 (71·4 %)	1 (100·0 %)	1 (100·0 %)	5 (45·5 %)	7 (87·5 %)	3 (100 %)
Renal replacement therapy	1 (5·3 %)	0 (0·0 %)	2 (22·2 %)	0 (0·0 %)	3 (42·9 %)	0 (0·0 %)	1 (100·0 %)	7 (63·6 %)	1 (12·5 %)	2 (66·7 %)
Multiple organ dysfunction	10 (52·6 %)	2 (28·6 %)	4 (44·4 %)	1 (50·0 %)	5 (71·4 %)	1 (100·0 %)	1 (100·0 %)	7 (63·6 %)	7 (87·5 %)	3 (100 %)
Palliative care	1 (5·3 %)	4 (57·1 %)	0 (0·0 %)	0 (0·0 %)	0 (0·0 %)	0 (0·0 %)	0 (0·0 %)	1 (9·1 %)	1 (12·5 %)	0 (0·0 %)
Cardiac arrest/ resuscitation	0 (0·0 %)	0 (0·0 %)	0 (0·0 %)	0 (0·0 %)	0 (0·0 %)	1 (100·0 %)	0 (0·0 %)	0 (0·0 %)	0 (0·0 %)	0 (0·0 %)

HIV, human immunodeficiency virus; ICU, intensive care unit

Supplementary references

1. The database of the German Federal Statistical Office. 2021. https://www-genesis.destatis.de/genesis/online (accessed 2 November 2021).
