# Supplementary material for: Evolving epidemiology of pneumocystis pneumonia: Findings from a longitudinal population-based study and a retrospective multi-center study in Germany
Source: Lancet Reg Health Eur. 2022 May 15;18:100400. doi: 10.1016/j.lanepe.2022.100400 (PMC9257643; doi:10.1016/j.lanepe.2022.100400)
Supplement: Supplementary file 3 [file mmc3.docx]

*This translation in German was submitted by the authors and we reproduce it as supplied. It has not been peer reviewed. Our editorial processes have only been applied to the original abstract in English, which should serve as reference for this manuscript.*

**Hintergrund:**

Die Pneumocystis-Pneumonie (PCP) ist eine opportunistische Infektion, die bei immunsupprimierten Patienten auftritt und oft lebensbedrohliche Verläufe nimmt. Bisher wurde angenommen, dass die Inzidenz und Mortalität der PCP weltweit sinken, allerdings fehlen aktuelle Studien.

**Methoden:**

Um die Epidemiologie sowie den klinischen Verlauf und die zugrundeliegenden Krankheiten von PCP-Patienten zu untersuchen, haben wir eine retrospektive Analyse aller Krankenhausfälle in Deutschland zwischen dem 01. Januar 2014 und dem 31. Dezember 2019 durchgeführt. Zudem haben wir während des gleichen Zeitraums in einer retrospektiven, multizentrischen Studie die PCP-Fälle an zwei großen deutschen Universitätskliniken anhand von Primärdaten analysiert.

**Resultate:**

Zwischen 2014 und 2019 konnte ein deutschlandweiter Anstieg der PCP-Inzidenz von 2·3 auf 2·6 pro 100,000 Einwohner beobachtet werden. Zudem kam es zu einem Anstieg der Mortalität um 19·2%. Während die PCP-Fälle und die damit assoziierte Mortalität in den Risikogruppen sanken, in denen bereits international definierte Leitlinien zur PCP-Prophylaxe bestehen (HIV, hämatologische Krebserkrankungen und Organtransplantierte), stellten wir einen deutlichen Anstieg der Fallzahlen und Todesfälle in den Risikogruppen mit soliden Tumoren, Autoimmunerkrankungen und Lungenerkrankungen fest. Der Einsatz von PCP-Chemoprophylaxe scheint hier unzureichend.

**Interpretation:**

Entgegen bisheriger Annahmen konnten wir eine Trendumkehr beobachten: Bevölkerungsweit kommt es zu einer Zunahme an PCP-Infektionen und damit verbundener Mortalität. Dies betrifft in besonderem Maße die Patientengruppen, welche typischerweise nicht mit einer PCP-Chemoprophylaxe behandelt werden. Unsere Daten zeigen den Bedarf an weiterführenden Studien auf und legen nahe, dass die bestehenden Leitlinien für die PCP-Chemoprophylaxe auf zusätzliche Risikogruppen ausgeweitet werden sollten.

**Finanzierung:**

Klinik für Nieren- und Hochdruckkrankheiten, Universitätsklinikum Schleswig-Holstein, Kiel.
